# Hypertriglyceridemic Waist Phenotype and Lipid Accumulation Product: Two Comprehensive Obese Indicators of Waist Circumference and Triglyceride to Predict Type 2 Diabetes Mellitus in Chinese Population

**DOI:** 10.1155/2020/9157430

**Published:** 2020-12-02

**Authors:** Minrui Xu, Mingtao Huang, Deren Qiang, Jianxin Gu, Yong Li, Yingzi Pan, Xingjuan Yao, Wenchao Xu, Yuan Tao, Yihong Zhou, Hongxia Ma

**Affiliations:** ^1^Wujin District Center for Disease Prevention and Control, Changzhou, Jiangsu, China; ^2^Department of Epidemiology, School of Public Health, Nanjing Medical University, Nanjing, China; ^3^Center for Global Health, School of Public Health, Nanjing Medical University, Nanjing, China; ^4^Department of Epidemiology, Jiangsu Key Lab of Cancer Biomarkers, Prevention and Treatment, Collaborative Innovation Center for Cancer Personalized Medicine, School of Public Health, Nanjing Medical University, Nanjing, China; ^5^Department of Prenatal Diagnosis, Nanjing Maternity and Child Health Care Hospital, Women's Hospital of Nanjing Medical University, Nanjing, China; ^6^Changzhou Center for Disease Prevention and Control, Changzhou, Jiangsu, China; ^7^Department of Medical Affairs, The Third Affiliated Hospital of Soochow University, The First People's Hospital of Changzhou, Changzhou, Jiangsu, China

## Abstract

**Purpose:**

To determine whether hypertriglyceridemic waist (HTGW) and high lipid accumulation product (LAP) preceded the incidence of type 2 diabetes mellitus (T2DM), and to investigate the interactions of HTGW and LAP with other components of metabolic syndrome on the risk of T2DM.

**Methods:**

A total of 15,717 eligible participants without baseline T2DM and aged 35 and over were included from a Chinese rural cohort. Cox proportional hazards regression models were used to estimate the association of HTGW and LAP with the incidence of T2DM, and the restricted cubic spline model was used to evaluate the dose-response association.

**Results:**

Overall, 867 new T2DM cases were diagnosed after 7.77 years of follow-up. Participants with HTGW had a higher hazard ratio for T2DM (hazard ratio (HR): 6.249, 95% confidence interval (CI): 5.199-7.511) after adjustment for potential confounders. The risk of incident T2DM was increased with quartiles 3 and 4 versus quartile 1 of LAP, and the adjusted HRs (95% CIs) were 2.903 (2.226-3.784) and 6.298 (4.911-8.077), respectively. There were additive interactions of HTGW (synergy index (SI): 1.678, 95% CI: 1.358-2.072) and high LAP (SI: 1.701, 95% CI: 1.406-2.059) with increased fasting plasma glucose (FPG) on the risk of T2DM. Additionally, a nonlinear (*P* nonlinear < 0.001) dose-response association was found between LAP and T2DM.

**Conclusion:**

The subjects with HTGW and high LAP were at high risk of developing T2DM, and the association between LAP and the risk of T2DM may be nonlinear. Our study further demonstrates additive interactions of HTGW and high LAP with increased FPG on the risk of T2DM.

## 1. Introduction

Type 2 diabetes mellitus (T2DM) is now a global health priority, and the International Diabetes Federation has predicted that the number of individuals with diabetes will increase from 425 million in 2017 to 700 million in 2045, with 80% of the disease burden in low- and middle-income countries [1]. With rapid economic development and nutritional transition, the rate of diabetes is increasing sharply in China, increasing from 2.5% in 1994 to 9.7% in 2008 among the Chinese population. It was estimated that approximately 92.4 million Chinese adults had diabetes [2] and that many were undiagnosed, untreated, or uncontrolled [3]. T2DM may reduce life expectancy by approximately 10 years [4] and increase the risk of microvascular and macrovascular complications in the general population, which results in a tremendous economic burden for society [5]. Therefore, it is important to identify and treat risk factors for T2DM.

Waist circumference (WC) and triglyceride (TG) levels are important components of metabolic syndrome, according to the definitions of the National Cholesterol Education Program-Third Adult Treatment Panel (NCEP-ATP III) [6] and International Diabetes Federation (IDF) [7]. However, increased WC cannot fully discriminate visceral adiposity from subcutaneous abdominal adiposity, and elevated triglyceride levels have been adopted as a marker of visceral obesity [8, 9]. Therefore, combining WC and TG can reflect visceral fat accumulation more comprehensively. Previous studies proposed two reliable comprehensive indicators to estimate visceral adiposity dysfunction base on WC and TG. One is hypertriglyceridemic waist (HTGW) phenotype, defined as elevated TG levels and enlarged WC, which was first proposed to be markers of atherosclerosis in 2000 [10]. Subsequent studies showed that HTGW was significantly associated with an increased risk of type 2 diabetes mellitus [11–16] and cardiovascular disease [17, 18]. The second is lipid accumulation product (LAP), calculated from WC and fasting TG levels, which was first proposed to recognize cardiovascular risk in 2005 [19]. Several studies indicated that LAP displayed a better predictive ability in metabolic syndrome, diabetes, and impaired fasting glucose as compared with common obesity indices [20–22]. Many studies have indicated that metabolic syndrome and its components were risk factors for T2DM [23, 24]. However, few studies have focused on the interaction effects of HTGW and LAP with other components of metabolic syndrome on the incident of T2DM, and no prior work has been done to explore the dose-response association between LAP and risk of T2DM.

In summary, this study intends to examine the independent and combined effects of HTGW and LAP with other components of metabolic syndrome on the incident of T2DM and to explore the dose-response association between LAP and risk of T2DM in a population-based prospective cohort study in China.

## 2. Methods

### 2.1. Study Cohort

**Figure 1:**
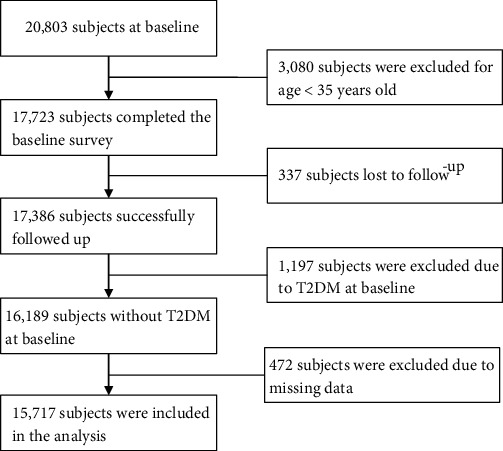
Flow chart of the study sample showing inclusion and exclusion of participants.

A population-based cohort study was established from June 2004 to September 2005 by a multistage and random cluster sampling method in Wujin District of Changzhou City, Jiangsu Province, China. First, we randomly selected 6 towns according to the economic development status of the 24 towns in the district. Subjects who were 35 years old or older and lived in their current residence for at least 5 years were eligible to participate. Informed consent was obtained from each participant before the survey. An in-person interview was conducted using a semistructured questionnaire to collect baseline data. The questionnaire covered demographic characteristics, socioeconomic status, personal behaviour, family history of selected diseases, physical activity, etc. Finally, a total of 20,803 subjects were selected for the cohort study, and 17,723 individuals aged 35 and over were included in this study. A total of 17,386 participants were followed up from July 2012 to August 2013. The same questionnaire interview, physical examination, and blood biochemical examination were performed at follow-up. We excluded people who had a diagnosis of T2DM at baseline (*n* = 1,197) and those who had missing data (*n* = 472) at baseline or at follow-up. A total of 15,717 participants (6,573 men and 9,144 women) were included in the analysis (Figure 1). The study protocol was approved by the ethics committee of Nanjing Medical University.

### 2.2. Measurements

Weight, height, waist circumference (WC), and blood pressure (BP) were measured following standardized protocols of the World Health Organization (WHO). Height was measured to the nearest 0.1 cm using a freestanding stadiometer, and weight was measured to the nearest 0.1 kg using a balance-beam scale with the participants wearing no shoes and lightweight clothing: body mass index (BMI) = weight (kg)/(height (metres))^2^. WC was measured with a tape measure to the nearest 0.1 cm at the midpoint between the lowest rib and the superior border of the iliac crest at the end of exhalation. Sitting blood pressure (BP) was recorded 3 times using a standard mercury sphygmomanometer after a rest of 15 minutes. Repeated measurements were required if the difference in blood pressure between two consecutive measurements was more than 10 mmHg, and the mean of the latter two values of blood pressure was used in the analysis.

### 2.3. Sample Collection and Laboratory Tests

A 5 ml blood sample was collected in the morning after at least 12 hours of fasting. The coagulated blood was then centrifuged at 3000 r/min for 10 minutes. The serum was used to measure the fasting plasma glucose (FPG), total cholesterol (TC), triglyceride (TG), and high-density lipoprotein-cholesterol (HDL-C) levels within 3 hours after blood collection. All biochemical markers were measured by using an OLYMPUS (C2734-Au640) automatic analyzer in the central laboratory of Changzhou Center for Disease Prevention and Control (CDC), which was authorized to perform laboratory tests according to the international quality standard ISO/IEC 17025. This study uses the method of Chen et al., and the method description partly reproduces their wording [25]. Assessment of quality control was performed by using quality control samples on a regular basis.

### 2.4. Definitions

Smoking and drinking behaviours were classified into two categories, current smokers/drinkers and noncurrent smokers/drinkers, according to information available at the time of the baseline survey. Current smokers were defined as persons who had smoked at least 1 cigarette per day during the preceding year; current drinkers were defined as individuals who consumed 3 or more alcoholic drinks per week for over 6 months [26]. Household income per month was divided into three levels: low (less than RMB 1000 yuan), moderate (1000-2999 yuan), and high (more than 3000 yuan). Family history of T2DM was defined as one or more first-degree relatives with T2DM. Metabolic syndrome (MS) was defined as central obesity (WC ≥ 90 cm in men and ≥80 cm in women) plus any two of the following: increased TG (TG ≥ 1.7 mmol/l (150 mg/dl)), reduced HDL-C (HDL‐C < 1.03 mmol/l (40 mg/dl) in men and <1.29 mmol/l (50 mg/dl) in women), increased BP (SBP ≥ 130 mmHg or DBP ≥ 85 mmHg or treatment of previously diagnosed hypertension), and increased FPG (FPG ≥ 5.6 mmol/l (100 mg/dl) or previously diagnosed type 2 diabetes) according to the new International Diabetes Federation definition for Chinese people [7]. Participants were classified into four groups by the TG level and WC: (i) NTNW, normal TG level and normal WC; (ii) NTGW, normal TG level and central obesity; (iii) HTNW, increased TG level and normal WC; and (iv) HTGW, increased TG level and central obesity. LAP was calculated as [WC (cm) − 65] × TG (mmol/l) for males and [WC (cm) − 58] × TG (mmol/l) for females [19]. It was classified into four groups (Q1, Q2, Q3, and Q4) by quartiles, and high LAP was determined based on the optimal cutoff point according to receiver operating characteristics (ROC) curve analysis. T2DM was defined according to current World Health Organization criteria using FPG ≥ 7.0 mmol/l) [27] or self-reported diabetes or the use of antidiabetic medication (oral agents or insulin) or a diagnosis of diabetes in the medical records excluding gestational diabetes mellitus, type 1 diabetes mellitus, or other types of diabetes. Finally, 867 cases (men: 330, women: 537) were identified during follow-up.

### 2.5. Statistical Analysis

A person-year (PY) was calculated as the time from the baseline investigation of the subjects to the date of T2DM diagnosis or the last time of follow-up. Multivariate Cox proportional hazards regression models were performed to estimate the hazard ratios (HRs) and 95% confidence intervals (CIs), with adjustments for age, sex, smoking status, drinking status, family history of T2DM, family income, and education. The best cutoff point was determined at the highest Youden index by using receiver operating characteristics (ROC) curve analysis. We then tested interactions on an additive scale by calculating the relative excess risk due to the interaction (RERI = RR_11_ − RR_10_ − RR_01_ + 1), the attributable proportion due to the interaction (AP = RERI/RR_11_), and the synergy index [SI = (RR_11_ − 1)/(RR_01_ − 1 + RR_10_ − 1)], based on the methods proposed by Andersson et al. [28]. RERI > 0, AP > 0, and SI > 1 indicate biological interactions. Both the point estimation and the 95% CI of RERI, AP, and SI were assessed using a method accounting for the asymmetric distribution of confidence limits for risk ratio [29]. A restricted cubic spline model with 4 knots at the 5th, 35th, 65th, and 95th percentiles was used to evaluate the dose-response association between LAP and risk of T2DM [30]. Statistical analyses were performed using the R project for statistical computing version 3.6.2 (Vienna, Austria, https://www.R-project.org/), and R package “epiR: Tools for the Analysis of Epidemiological Data” version 1.0-14 (Mark Stevenson et al. https://CRAN.R-project.org/package=epiR) was used to calculate the additive interactions. Statistical significance was defined at the level of *P* < 0.05 (two-tailed).

## 3. Results

### 3.1. Baseline Characteristics of the Study Participants

A total of 15,717 participants (6,573 men and 9,144 women) were included in the analysis, and the median (interquartile range) follow-up time was 7.77 (7.05-8.05) years. Furthermore, 867 new T2DM cases (330 men and 537 women) were identified during 114,221.61 person-years (PYs) of follow-up, and the incidence rate was 7.591 (6.931 in men and 8.062 in women) per 1000 PYs. At baseline, 8,615 subjects (54.81%) were in the NTNW group, 3,454 (21.98%) were in the NTGW group, 1,593 (10.14%) were in the HTNW group, and 2,055 (13.08%) were in the HTGW group. The baseline characteristics by gender are shown in Table 1. Females had higher BMI, TC, HDL, FPG, and LAP and lower age, SBP, DBP, and WC than males (*P* < 0.05). In addition, males and females differed in the distribution of education, physical activity, income level, smoking, alcohol consumption, history of hypertension, family history of diabetes, and HTGW phenotype (*P* < 0.05).

### 3.2. Hazard Ratios of T2DM according to Phenotype Groups and Quartile of LAP

The incidence rates of T2DM in the NTNW, NTGW, HTNW, and HTGW groups were 3.347, 10.812, 8.192, and 19.958 per 1000 PYs, respectively. And the incidence rates in the Q1, Q2, Q3, and Q4 of LAP groups were 2.731, 3.082, 7.984, and 16.949 per 1000 PYs, respectively. The Cox proportional hazards regression model showed that the HRs (95% CIs) of T2DM in the NTGW, HTNW, and HTGW groups were 3.422 (2.833-4.135), 2.542 (1.994-3.240), and 6.249 (5.199-7.511), respectively, compared to the NTNW group after adjustment for age, sex, smoking status, drinking status, physical activity, family history of T2DM, family income, and education. In the groups described above, the respective HRs (95% CIs) were 4.071 (3.014-5.499), 2.205 (1.584-3.070), and 5.976 (4.493-7.946) in men and 3.214 (2.504-4.126), 3.049 (2.127-4.370), and 6.299 (4.892-8.111) in women after adjustment for potential confounders described above (Table 2). The HRs (95% CIs) of T2DM in the Q2, Q3, and Q4 of LAP groups were 1.169 (0.857-1.595), 2.903 (2.226-3.784), and 6.298 (4.911-8.077), respectively, compared to the Q1 group after adjustment for potential confounders. The adjusted HRs (95% CIs) in Q2, Q3, and Q4 groups were 1.123 (0.719-1.752), 1.839 (1.230-2.748), and 4.773 (3.324-6.854) in men and 1.633 (1.073-2.485), 4.150 (2.865-6.013), and 8.063 (5.645-11.516) in women (Table 2).

### 3.3. Associations of Metabolic Syndrome Components and the Incidence of T2DM

The optimum cutoff point for LAP was 27.90 according to ROC curve analysis. The incidence rate of T2DM in subjects with high LAP, HTGW, central obesity, increased BP, increased FPG, increased TG, reduced HDL-C, and MS were 14.710, 19.958, 14.192, 11.075, 23.668, 14.715, 9.212, and 21.402 per 1000 PYs, respectively. The Cox proportional hazards model showed that HRs (95% CIs) of T2DM in subjects with high LAP, HTGW, central obesity, increased BP, increased FPG, increased TG, reduced HDL-C, and MS were 4.094 (3.530-4.755), 3.387 (2.929-3.916), 3.671 (3.163-4.260), 2.111 (1.820-2.448), 5.532 (4.829-6.337), 2.719 (2.375-3.114), 1.209 (1.034-1.413), and 4.224 (3.671-4.86) after adjustment for age, sex, smoking status, drinking status, physical activity, family history of T2DM, family income, and education. The HRs of T2DM increased gradually with the number of MS components (*P* for trend test < 0.001) (Table 3).

### 3.4. Additive Interaction Effects of HTGW and LAP with Other Components of Metabolic Syndrome on the Incidence of T2DM

Based on the results shown in Table 3, we further investigated the effects of the interaction of HTGW and high LAP with other components of MS (increased BP, increased impaired fasting glucose (IFG), and reduced HDL-C) on the risk of T2DM. The results showed that there was an additive interaction between HTGW and increased FPG, and the SI (95% CI) was 1.678 (1.358-2.072), suggesting that the risk of T2DM in HTGW subjects with increased FPG was 1.678 times as high as the sum of risks in participants exposed to a single risk factor alone. There were no significant additive interactions between HTGW and raised BP (SI: 1. 067, 95% CI: 0.799-1.425) and reduced LDL_C (SI: 0.761, 95% CI: 0.534-1.084) after adjusting for potential confounders (Table 4). Additionally, additive interactions were found between high LAP and increased FPG (SI: 1.701, 95% CI: 1.406-2.059) and increased BP (SI: 1.298, 95% CI: 1.045-1.611), suggesting that the risk of T2DM in subjects with high LAP and either increased FPG or increased BP was 1.701 and 1.298 times as high, respectively, as the sum of risks in participants with a single risk factor alone. The interaction between LAP and reduced LDL_C (SI: 0.871, 95% CI: 0.672-1.129) was not significant (Table 5).

### 3.5. Dose-Response Association between LAP and Risk of T2DM

**Figure 2:**
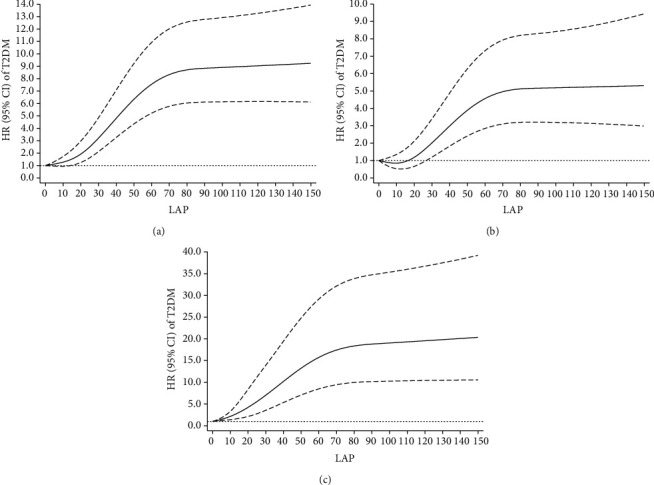
Nonlinear associations of lipid accumulation product (LAP) and risk of type 2 diabetes mellitus (T2DM) by using restricted cubic splines with 4 knots in all (a), men (b), and women (c). Adjustment was for age, sex, smoking status, drinking status, physical activity, family history of T2DM, family income, and education. HR: hazard ratio; CI: confidence interval; T2DM: type 2 diabetes mellitus.

A restricted cubic spline model with 4 knots at the 5th, 35th, 65th, and 95th percentiles was used to evaluate the dose-response association between LAP and risk of T2DM. The spline suggested a nonlinear relationship (*P* nonlinear = 0.001) between LAP and risk of T2DM after adjustment for age, sex, smoking status, drinking status, physical activity, family history of T2DM, family income, and education. The adjusted HR for T2DM significantly increased with increasing LAP, but it tends towards stability when the LAP is over than about 80, and similar phenomena were observed in both men and women (Figure 2).

## 4. Discussion

In this study, the incidence rate of T2DM was 7.591 (6.931 in men and 8.062 in women) per 1000 PYs after a median (interquartile range) follow-up time of 7.77 (7.05-8.05) years. Central obesity, increased blood pressure, increased triglyceride level, increased fasting glucose, and metabolic syndrome were all associated with the incidence of T2DM. The Cox proportional hazards model showed a significantly increased risk of T2DM development in the HTGW and high LAP groups after adjustment for age, sex, smoking status, drinking status, physical activity, family history of T2DM, family income, and education. Our study further demonstrates the additive interaction effects of HTGW and high LAP with increased FPG, and the risks of T2DM were significantly higher than the sum of risks in participants exposed to a single risk factor alone. Additionally, a nonlinear dose-response association was found between LAP and T2DM.

Elevated TG and enlarged WC, known as the HTGW phenotype, were first proposed in 2000 [10]. As a specific type of metabolic disorder, HTGW is widely considered to be associated with hypertension [31], T2DM [11–16], atherosclerosis [10], and cardiovascular disease [17, 18]. The prevalence of the HTGW phenotype was 13.08% in this study. A meta-analysis of 25 studies showed that the prevalence of HTGW ranged from 4% to 47%, and the pooled prevalence was 18% (95% CI, 13%-23%). There was a strong association between HTGW and T2DM, with ORs ranging from 2.8 to 9.6 and pooled ORs of 4.18 (95% CI, 3.55-4.92) [32]. According to a recent meta-analysis, the pooled OR for diabetes related to HTGW phenotype was 2.89 (95% CI: 1.97-4.25) in cohort studies and 2.66 (95% CI: 2.35-3.01) in cross-sectional studies [33]. Some studies concluded that HTGW may be an alternative to MS for detecting the population at risk for T2DM and CVD, especially in young individuals who do not fulfill the criteria for MS [34]. Our study also showed that the incidence rate of T2DM was highest in the HTGW group (23.525 per 1000 PYs). The Cox proportional hazards regression model showed that the HRs and 95% CIs of T2DM in the NTGW, HTNW, and HTGW groups were 3.422 (2.833-4.135), 2.542 (1.994-3.240), and 6.249 (5.199-7.511), respectively, compared to the NTNW group after adjustment for age, sex, smoking status, drinking status, physical activity, family history of T2DM, family income, and education, and similar results were found in both men and women. The mechanism behind the association between the HTGW phenotype and T2DM remains unclear. Previous findings suggested that enlarged waist circumference was associated with increased intra-abdominal fats, which has been found to be an independent risk factor for T2DM [35]. On the other hand, high intra-abdominal fat increases the release of free fatty acids in circulation, which can induce insulin resistance and hyperinsulinaemia [36]. The reported predictive effects of NTGW and HTNW on T2DM are inconsistent; several cross-sectional studies have shown that HTNW has a stronger correlation with T2DM than does NTGW [37, 38]. However, cohort studies confirmed that the predictive effect of NTGW on T2DM was better for subjects with HTNW [15, 16]. According to a recent meta-analysis, the pooled OR for diabetes was 2.37 (95% CI: 2.04-2.75) in HTNW subjects and 2.66 (95% CI: 2.11-3.34) in NTGW subjects compared with NTNW subjects [33]. Insulin resistance may be the mechanism that explains this phenomenon; cohort [16] and cross-sectional studies [39, 40] have shown that insulin resistance is more marked in NTHW subjects than in HTNW subjects.

LAP is calculated from WC and fasting TG levels, which has a theoretical basis in reflecting the accumulation of visceral fat [19]. Several studies indicated that LAP displayed a better predictive ability in metabolic syndrome, diabetes, and impaired fasting glucose as compared with common obesity indices [20–22]. But some studies indicated that high LAP was not associated with an increased risk of T2DM [41], and LAP may not be an inexpensive tool for predicting T2DM [42]. In this study, LAP significantly associates with T2DM risk in both men and women; the HRs in the fourth quartile groups were 4.773 and 8.063 in comparison with the first quartile of LAP, respectively. Similar results were found in the Korean, Chinese, and Japanese populations [42–44]. The potential mechanism behind the association between LAP and T2DM can be explained by insulin resistance. The Framingham Heart Study demonstrated that visceral adipose tissue was a stronger correlate of insulin resistance [45]. As an indicator of visceral adipose tissue, LAP was a useful predictor of insulin resistance [46]. To our knowledge, the dose-response association between LAP and risk of T2DM has not been explored before. In this study, a nonlinear dose-response association was found between LAP and T2DM after adjustment for age, sex, smoking status, drinking status, physical activity, family history of T2DM, family income, and education, and the adjusted HR for T2DM significantly increased with increasing LAP, but it tends towards stability when the LAP is over than about 80. However, the reason of this phenomenon is not fully clear; more further studies are needed to explore the dose-response association between LAP and T2DM risk.

The associations of both HTGW and LAP with T2DM were stronger in women than in men. A meta-analysis by Zhou et al. [47] showed that the pooled OR for diabetes related to HTGW phenotype was higher for females (3.16 (95% CI: 2.51-3.97)) than for males (2.65 (95% CI: 2.17-3.25)), and a similar result was found in this study; the adjusted HR (95% CI) of HTGW was 5.976 (4.493-7.946) in men and 6.299 (4.892-8.111) in women. A Chinese cohort study indicated that the adjusted HR (95% CI) for the fourth quartile of LAP was 5.02 (2.85-8.85) in men and 6.49 (3.48-12.12) in women as compared with the first quartile [42]. A cross-sectional study of 215,651 Chinese adults found that the predictive accuracy of LAP was higher for women than for men [22]. In our study, the adjusted HR (95% CI) of T2DM in Q4 of LAP was higher for women 8.063 (5.645-11.516) than for men 6.298 (4.911-8.077) as compared with the first quartile. However, the detailed mechanism of this effect is still not clear; we hypothesized that this might be due to the fact that the anthropometric relationship with T2DM is stronger in women than in men [48, 49]. The previous findings are consistent with the present study, indicating that women with HTGW and high LAP had a higher risk of T2DM than men.

The relationship between metabolic syndrome and its components and diabetes has been well examined in previous studies [23, 24]. A study from China indicated that the adjusted relative risk of developing T2DM in individuals with MS was 2.3 times greater than that in individuals without MS at baseline and that the relative risk was approximately 12.0 times greater when five components coexisted [50]. In the present study, the adjusted HR in subjects with MS was 4.224, and the HRs increased gradually with the number of MS components. A meta-analysis with 20 studies showed that there was a strong association between abdominal obesity and the incidence of type 2 diabetes, and the pooled odds ratio was 2.14 [51]. Our study indicated an association between T2DM and abdominal obesity, and the HR was 3.671 after adjusting for diverse confounding variables. IFG was found to be an excellent predictor of T2DM. Japanese [52] and American [53] studies suggest that IFG is a major risk factor compared with other components of metabolic syndrome. Our results also demonstrated that IFG was a powerful risk factor for T2DM compared with other metabolic syndrome components. The HR of T2DM was 5.532, which was similar to a study in China [50]. Hypertension and even elevated blood pressure (SBP ≥ 130 mmHg or DBP ≥ 85 mmHg) are known to contribute to the development of T2DM directly or indirectly [54]. A meta-analysis indicated that the pooled OR of hypertension on T2DM was 2.73 (2.25-3.36) in Chinese [47]. Our study also showed that elevated blood pressure was a risk factor for T2DM, and the HR was 2.534 after adjusting for diverse confounding variables.

The interaction effect of HTGW and LAP with other MS components on the risk of T2DM has not been well explored in previous studies. Only one study in China demonstrated an additive interaction effect between HTGW and increased FPG on the risk of T2DM, and the SI was 2.66 [55]. In this study, additive interaction effects of HTGW and LAP with increased PFG on the risk of T2DM were found; the SIs (95% CI) were 1.678 (95% CI: 1.358-2.072) and 1.701 (95% CI: 1.406-2.059), respectively, which suggested that the risk of T2DM was significantly higher than the sum of risks in participants exposed to a single risk factor alone. However, there were no multiplicative interactions among factors. Several studies have demonstrated that impaired fasting glucose was closely related to insulin resistance, obesity, and lipid metabolism disorders, which form a vicious circle of causality [56, 57]. Therefore, the potential mechanism that HTGW, LAP, and IFG jointly cause insulin resistance can explain the additive interaction between HTGW and IFG on the risk of T2DM.

The limitations of this study should be considered. First, the diagnosis of T2DM was determined by self-report and FPG, but a lack of OGTT (oral glucose tolerance test) and determination of glycosylated haemoglobin may have caused some people with diabetes to not be identified. Second, there were more women (58.18%) than men (41.82%); however, gender was adjusted in the analysis as a confounding factor or as a stratified variable. Third, we could not evaluate the potential effect of dietary intake on T2DM, which is an important risk factor for diabetes [58]. Finally, an additional limitation was that we did not consider the concurrent use of lipid-modifying agents at baseline. This might lead to an underestimation of the prevalence of HTGW phenotypes; furthermore, it is known that the use of lipid-modifying agents can induce the development of T2DM [59], and this might lead to overestimation of the association between HTGW and T2DM risk. However, considering the low treatment rate of dyslipidaemia in China [60], we hypothesized that the use of lipid-modifying agents is not likely to have a significant impact on the validity of our results.

## 5. Conclusions

In conclusion, the results from this Chinese prospective cohort study suggested HTGW and LAP were effective predictors of T2DM, and the association between LAP and the risk of T2DM may be nonlinear. Moreover, this study further demonstrated additive interactions of HTGW and LAP with increased FPG on the risk of T2DM. Because measurements of WC and TG are relatively simple to perform in a clinical setting, using the HTGW phenotype and LAP to identify individuals at high risk of T2DM has important public health implications for early prevention and treatment, especially in individuals with HTGW, high LAP, and increased FPG.

## Figures and Tables

**Table 1:** Baseline characteristics of study participants by gender.

Variables	All (*n* = 15,717)	Males (*n* = 6,573)	Females (*n* = 9,144)	*P* values^∗^
Age (years)	52.70 ± 11.58	52.94 ± 11.31	52.53 ± 11.76	0.031
Education, *n* (%)				
No education	1,913 (12.17)	311 (4.73)	1,602 (17.52)	<0.001
Primary school	5,535 (35.22)	2,277 (34.64)	3,258 (35.63)	
Middle school	6,677 (42.48)	3,157 (48.03)	3,520 (38.5)	
High school	1,391 (8.85)	715 (10.88)	676 (7.39)	
College and above	75 (0.48)	64 (0.97)	11 (0.12)	
Unknown	126 (0.80)	49 (0.75)	77 (0.84)	
Physical activity, *n* (%)				
Low	6,483 (41.25)	2,294 (34.90)	4,189 (45.81)	<0.001
Moderate	7,428 (47.26)	3,188 (48.50)	4,240 (46.37)	
High	1,651 (10.50)	1,049 (15.96)	602 (6.58)	
Unknown	155 (0.99)	42 (0.64)	113 (1.24)	
Income, *n* (%)				
Low	2,038 (12.97)	879 (13.37)	1,159 (12.67)	0.015
Moderate	13,027 (82.88)	5,406 (82.25)	7,621 (83.34)	
High	500 (3.18)	235 (3.58)	265 (2.9)	
Unknown	152 (0.97)	53 (0.81)	99 (1.08)	
Current smokers, *n* (%)	4,210 (26.79)	4,124 (62.74)	86 (0.94)	<0.001
Current drinkers, *n* (%)	3,649 (23.22)	3,282 (49.93)	367 (4.01)	<0.001
Hypertension, *n* (%)	5,237 (33.32)	2,356 (35.84)	2,881 (31.51)	<0.001
Family history of T2DM, *n* (%)	891 (5.67)	342 (5.20)	549 (6.00)	0.037
SBP (mmHg)	124.93 ± 20.03	125.74 ± 19.00	124.35 ± 20.72	<0.001
DBP (mmHg)	79.84 ± 10.70	81.43 ± 10.69	78.68 ± 10.55	<0.001
FPG (mmol/l)	5.01 ± 0.68	4.96 ± 0.70	5.04 ± 0.67	<0.001
TC (mmol/l)	4.39 ± 1.02	4.31 ± 1.00	4.44 ± 1.04	<0.001
TG (mmol/l)	1.09 (0.76-1.64)	1.07 (0.74-1.65)	1.11 (0.78-1.63)	0.076
HDL-C (mmol/l)	1.52 ± 0.48	1.50 ± 0.48	1.53 ± 0.48	<0.001
BMI (kg/m^2^)	23.32 ± 3.30	23.04 ± 3.06	23.52 ± 3.45	<0.001
WC (cm)	79.83 ± 9.68	81.13 ± 9.44	78.90 ± 9.75	<0.001
LAP	20.15 (9.90-38.49)	16.73 (7.68-34.75)	22.40 (11.78-40.92)	<0.001
HTGW phenotype				
NTNW	8,615 (54.81)	4,322 (65.75)	4,293 (46.95)	<0.001
NTGW	3,454 (21.98)	683 (10.39)	2,771 (30.30)	
HTNW	1,593 (10.14)	942 (14.33)	651 (7.12)	
HTGW	2,055 (13.08)	626 (9.52)	1429 (15.63)	

BMI: body mass index; WC: waist circumference; SBP: systolic blood pressure; DBP: diastolic blood pressure; FPG: fast plasma glucose; TC: total cholesterol; TG: triglyceride; HDL-C: high-density lipoprotein cholesterol; LAP: lipid accumulation product; NTNW: normal TG level and normal waist; NTGW: normal TG level and central obesity; HTNW: increased TG level and normal WC; HTGW: increased TG level and central obesity. Values are expressed as mean ± SD or number (%); TG and LAP were described by median and interquartile range and were analyzed after logarithmic transformation. ^∗^Comparison between different genders.

**Table 2:** Hazard ratios for the incidence of T2DM according to the four phenotype groups and quartile of LAP.

	*N*	T2DM cases	Incidence density	Unadjusted model		Multivariable adjusted model^#^	
				HR (95% CI)	*P* values	HR (95% CI)	*P* values
*HTGW phenotype*							
Total							
NTNW	8,615	211	3.347	1.000 (ref)	—	1.000 (ref)	—
NTGW	3,454	269	10.812	3.331 (2.781-3.989)	<0.001	3.422 (2.833-4.135)	<0.001
HTNW	1,593	96	8.192	2.493 (1.959-3.174)	<0.001	2.542 (1.994-3.240)	<0.001
HTGW	2,055	291	19.958	6.301 (5.277-7.523)	<0.001	6.249 (5.199-7.511)	<0.001
Men							
NTNW	4,322	116	3.700	1.000 (ref)	—	1.000 (ref)	—
NTGW	683	71	14.462	4.015 (2.988-5.394)	<0.001	4.071 (3.014-5.499)	<0.001
HTNW	942	52	7.496	2.037 (1.469-2.825)	<0.001	2.205 (1.584-3.070)	<0.001
HTGW	626	91	20.604	5.733 (4.357-7.544)	<0.001	5.976 (4.493-7.946)	<0.001
Women							
NTNW	4,293	95	2.997	1.000 (ref)	—	1.000 (ref)	—
NTGW	2,771	198	9.915	3.410 (2.670-4.356)	<0.001	3.214 (2.504-4.126)	<0.001
HTNW	651	44	9.202	3.171 (2.218-4.533)	<0.001	3.049 (2.127-4.370)	<0.001
HTGW	1,429	200	19.677	6.978 (5.466-8.909)	<0.001	6.299 (4.892-8.111)	<0.001
*Quartile of LAP* ^∗^							
Total							
Q1 (LAP < 9.9)	3,823	76	2.731	1.000 (ref)	—	1.000 (ref)	—
Q2 (LAP 9.9-20.1)	3,848	87	3.082	1.153 (0.848-1.569)	0.364	1.169 (0.857-1.595)	0.325
Q3 (LAP 20.2-38.4)	3,839	224	7.984	2.972 (2.291-3.856)	<0.001	2.903 (2.226-3.784)	<0.001
Q4 (LAP ≥ 38.5)	3,837	465	16.949	6.523 (5.118-8.314)	<0.001	6.298 (4.911-8.077)	<0.001
Men							
Q1 (LAP < 7.7)	1,582	39	3.425	1.000 (ref)	—	1.000 (ref)	—
Q2 (LAP 7.7-16.6)	1,581	41	3.593	1.075 (0.693-1.666)	0.747	1.123 (0.719-1.752)	0.610
Q3 (LAP 16.7-34.7)	1,580	69	5.928	1.743 (1.177-2.582)	0.006	1.839 (1.230-2.748)	0.003
Q4 (LAP ≥ 34.8)	1,583	172	15.096	4.535 (3.203-6.421)	<0.001	4.773 (3.324-6.854)	<0.001
Women							
Q1 (LAP < 11.8)	2,255	35	2.100	1.000 (ref)	—	1.000 (ref)	—
Q2 (LAP 11.8-22.3)	2,255	59	3.550	1.718 (1.131-2.610)	0.011	1.633 (1.073-2.485)	0.022
Q3 (LAP 22.4-40.8)	2,254	150	9.164	4.480 (3.101-6.474)	<0.001	4.150 (2.865-6.013)	<0.001
Q4 (LAP ≥ 40.9)	2,257	287	17.882	9.058 (6.377-12.867)	<0.001	8.063 (5.645-11.516)	<0.001

Incidence density, per 1000 person-years; NTNW: normal TG level and normal waist; NTGW: normal TG level and central obesity; HTNW: increased TG level and normal WC; HTGW: increased TG level and central obesity. ^#^Adjusted for age, smoking status, drinking status, physical activity, family history of T2DM, family income, and education; adjusted for sex in the total population at the same time. ^∗^370 (247 for men and 123 for women) subjects were excluded because of LAP less than 0.

**Table 3:** Hazard ratios for the incidence of T2DM in different metabolic groups.

	*N*	T2DM cases	Incidence density	Unadjusted model		Multivariable adjusted model^#^	
				HR (95% CI)	*P* values	HR (95% CI)	*P* values
High LAP^∗^							
No	9,695	255	3.593	1.000 (ref)	—	1.000 (ref)	—
Yes	5,652	597	14.710	4.243 (3.660-4.913)	<0.001	4.094 (3.530-4.755)	<0.001
HTGW							
No	13,662	576	5.781	1.000 (ref)	—	1.000 (ref)	—
Yes	2,055	291	19.958	3.611 (3.136-4.158)	<0.001	3.387 (2.929-3.916)	<0.001
Central obesity							
No	10,208	307	4.106	1.000 (ref)	—	1.000 (ref)	—
Yes	5,509	560	14.192	3.585 (3.119-4.121)	<0.001	3.671 (3.163-4.260)	<0.001
Increased BP							
No	8,705	311	4.858	1.000 (ref)	—	1.000 (ref)	—
Yes	7,012	556	11.075	2.281 (1.986-2.621)	<0.001	2.111 (1.820-2.448)	<0.001
Increased FPG							
No	12,943	403	4.259	1.000 (ref)	—	1.000 (ref)	—
Yes	2,774	464	23.668	5.747 (5.028-6.567)	<0.001	5.532 (4.829-6.337)	<0.001
Increased TG							
No	12,069	480	5.459	1.000 (ref)	—	1.000 (ref)	—
Yes	3,648	387	14.715	2.777 (2.429-3.175)	<0.001	2.719 (2.375-3.114)	<0.001
Reduced HDL-C							
No	12,396	640	7.144	1.000 (ref)	—	1.000 (ref)	—
Yes	3,321	227	9.212	1.194 (1.026-1.390)	0.022	1.209 (1.034-1.413)	0.017
MS							
No	13,120	473	4.937	1.000 (ref) (ref)	—	1.000 (ref) (ref)	—
Yes	2,597	394	21.402	4.419 (3.866-5.052)	<0.001	4.224 (3.671-4.860)	<0.001
Number of MS components							
0	4,250	54	1.726	1.000 (ref)	—	1.000 (ref)	—
1	4,909	125	3.496	2.014 (1.463-2.771)	<0.001	1.996 (1.441-2.763)	<0.001
2	3,508	248	9.754	5.704 (4.249-7.656)	<0.001	5.728 (4.238-7.741)	<0.001
3	2,032	239	16.381	9.589 (7.137-12.884)	<0.001	9.437 (6.962-12.793)	<0.001
4	847	149	24.809	14.423 (10.564-19.691)	<0.001	14.846 (10.774-20.458)	<0.001
5	171	52	44.734	26.219 (17.914-38.372)	<0.001	24.652 (16.612-36.583)	<0.001

Incidence density, per 1000 person-years; BP: blood pressure; FPG: fast plasma glucose; TG: triglyceride; HDL-C: high-density lipoprotein cholesterol; MS: metabolic syndrome. ^#^Adjusted for age, sex, smoking status, drinking status, physical activity, family history of T2DM, family income, and education. ^∗^Grouped by optimal cutoff value (LAP = 27.90) calculating through ROC curve analysis, 370 (247 for men and 123 for women) subjects were excluded because of LAP less than 0.

**Table 4:** Interactions between HTGW and other components of metabolic syndrome on the risk of T2DM.

HTGW	MS components	*N*	T2DMcases	Incidence density	Unadjusted model		Multivariable adjusted model^#^	
					HR (95% CI)	*P* value	HR (95% CI)	*P* value
HTGW	Increased BP							
No	No	8,038	234	3.953	1.000 (ref)	—	1.000 (ref)	—
Yes	No	667	77	15.956	4.180 (3.231-5.407)	<0.001	4.013 (3.091-5.210)	<0.001
No	Yes	5,624	342	8.455	2.124 (1.799-2.508)	<0.001	1.970 (1.653-2.348)	<0.001
Yes	Yes	1,388	214	21.937	5.808 (4.825-6.991)	<0.001	5.251 (4.329-6.369)	<0.001
					SI (95% CI): 1.117 (0.846-1.475)		SI (95% CI): 1.067 (0.799-1.425)	
					AP (95% CI): 0.087 (-0.12-0.294)		AP (95% CI): 0.051 (-0.169-0.271)	
					REEI (95% CI): 0.504 (-0.734-1.742)		REEI (95% CI): 0.268 (-0.907-1.442)	
HTGW	Increased FPG							
No	No	11,581	296	3.494	1.000 (ref)	—	1.000 (ref)	—
Yes	No	1,362	107	10.815	3.200 (2.565-3.992)	<0.001	3.017 (2.409-3.778)	<0.001
No	Yes	2,081	280	18.770	5.500 (4.671-6.476)	<0.001	5.286 (4.478-6.238)	<0.001
Yes	Yes	693	184	39.258	12.202 (10.15-14.669)	<0.001	11.573 (9.588-13.971)	<0.001
					SI (95% CI): 1.672 (1.36-2.056)		SI (95% CI): 1.678 (1.358-2.072)	
					AP (95% CI): 0.369 (0.252-0.486)		AP (95% CI): 0.369 (0.251-0.488)	
					REEI (95% CI): 4.502 (2.489-6.516)		REEI (95% CI): 4.271 (2.33-6.212)	
HTGW	Reduced HDL-C							
No	No	11,031	451	5.632	1.000 (ref)	—	1.000 (ref)	—
Yes	No	1,365	189	19.886	3.858 (3.255-4.573)	<0.001	3.697 (3.108-4.397)	<0.001
No	Yes	2,631	125	6.389	1.058 (0.868-1.290)	0.575	1.117 (0.913-1.366)	0.284
Yes	Yes	690	102	20.092	3.331 (2.686-4.130)	<0.001	3.140 (2.514-3.923)	<0.001
					SI (95% CI): 0.799 (0.567-1.126)		SI (95% CI): 0.761 (0.534-1.084)	
					AP (95% CI): -0.176 (-0.465-0.113)		AP (95% CI): -0.214 (-0.516-0.088)	
					REEI (95% CI): -0.586 (-1.468-0.296)		REEI (95% CI): -0.673 (-1.528-0.182)	

Incidence density, per 1000 person-years; HTGW: increased TG level and central obesity; BP: blood pressure; FPG: fast plasma glucose; HDL-C: high-density lipoprotein cholesterol; MS: metabolic syndrome; SI: synergy index; AP: attributable proportion due to the interaction; RERI: relative excess risk due to the interaction. ^#^Adjusted for age, sex, smoking status, drinking status, physical activity, family history of T2DM, family income, and education.

**Table 5:** Interactions between high LAP and other components of metabolic syndrome on the risk of T2DM.

High LAP^∗^	MS components	*N*	T2DM cases	Incidence density	Unadjusted model		Multivariable adjusted model^#^	
					HR (95% CI)	*P* value	HR (95% CI)	*P* value
High LAP	Increased BP							
No	No	6,210	123	2.684	1.000 (ref)	—	1.000 (ref)	—
Yes	No	2,251	180	10.961	4.195 (3.335-5.276)	<0.001	4.169 (3.306-5.257)	<0.001
No	Yes	3,485	132	5.252	1.931 (1.510-2.468)	<0.001	1.799 (1.395-2.320)	<0.001
Yes	Yes	3,401	417	17.259	6.651 (5.440-8.133)	<0.001	6.15 (4.987-7.584)	<0.001
					SI (95% CI): 1.370 (1.110-1.690)		SI (95% CI): 1.298 (1.045-1.611)	
					AP (95% CI): 0.229 (0.099-0.360)		AP (95% CI): 0.192 (0.052-0.332)	
					REEI (95% CI): 1.526 (0.577-2.475)		REEI (95% CI): 1.182 (0.259-2.105)	
High LAP	Increased FPG							
No	No	8,497	134	2.152	1.000 (ref)	—	1.000 (ref)	—
Yes	No	4,105	257	8.601	4.088 (3.318-5.038)	<0.001	4.015 (3.25-4.96)	<0.001
No	Yes	1,198	121	13.911	6.464 (5.055-8.265)	<0.001	6.33 (4.936-8.118)	<0.001
Yes	Yes	1,547	340	31.760	15.911 (13.026-19.434)	<0.001	15.198 (12.393-18.638)	<0.001
					SI (95% CI): 1.744 (1.445-2.104)		SI (95% CI): 1.701 (1.406-2.059)	
					AP (95% CI): 0.400 (0.298-0.502)		AP (95% CI): 0.385 (0.279-0.491)	
					REEI (95% CI): 6.359 (4.063-8.655)		REEI (95% CI): 5.853 (3.620-8.086)	
High LAP	Reduced HDL-C							
No	No	8,101	215	3.640	1.000 (ref)	—	1.000 (ref)	—
Yes	No	3,961	412	14.654	4.303 (3.649-5.075)	<0.001	4.203 (3.554-4.970)	0.706
No	Yes	1,594	40	3.360	0.869 (0.620-1.218)	0.415	0.937 (0.666-1.316)	<0.001
Yes	Yes	1,691	185	14.837	3.832 (3.147-4.665)	<0.001	3.735 (3.051-4.573)	<0.001
					SI (95% CI): 0.893 (0.693-1.150)		SI (95% CI): 0.871 (0.672-1.129)	
					AP (95% CI): -0.089 (-0.296-0.118)		AP (95% CI): -0.108 (-0.322-0.106)	
					REEI (95% CI): -0.341 (-1.105-0.424)		REEI (95% CI): -0.404 (-1.171-0.363)	

Incidence density, per 1000 person-years; HTGW: increased TG level and central obesity; BP: blood pressure; FPG: fast plasma glucose; HDL-C: high-density lipoprotein cholesterol; MS: metabolic syndrome; SI: synergy index; AP: attributable proportion due to the interaction; RERI: relative excess risk due to the interaction. ^#^Adjusted for age, sex, smoking status, drinking status, physical activity, family history of T2DM, family income, and education. ^∗^Grouped by optimal cutoff value (LAP = 27.90) calculating through ROC curve analysis, 370 (247 for men and 123 for women) subjects were excluded because of LAP less than 0.

## Data Availability

The datasets used to support this study are not freely available in view of participants' privacy protection.
